# Resolving the neural dynamics of visual and auditory scene processing in the human brain: a methodological approach

**DOI:** 10.1098/rstb.2016.0108

**Published:** 2017-02-19

**Authors:** Radoslaw Martin Cichy, Santani Teng

**Affiliations:** 1 Department of Education and Psychology, Free University Berlin, Berlin, Germany; 2 Computer Science and Artificial Intelligence Laboratory, Massachusetts Institute of Technology, Cambridge, MA, USA

**Keywords:** scene perception, multivariate pattern classification, deep neural networks, representational similarity analysis

## Abstract

In natural environments, visual and auditory stimulation elicit responses across a large set of brain regions in a fraction of a second, yielding representations of the multimodal scene and its properties. The rapid and complex neural dynamics underlying visual and auditory information processing pose major challenges to human cognitive neuroscience. Brain signals measured non-invasively are inherently noisy, the format of neural representations is unknown, and transformations between representations are complex and often nonlinear. Further, no single non-invasive brain measurement technique provides a spatio-temporally integrated view. In this opinion piece, we argue that progress can be made by a concerted effort based on three pillars of recent methodological development: (i) sensitive analysis techniques such as decoding and cross-classification, (ii) complex computational modelling using models such as deep neural networks, and (iii) integration across imaging methods (magnetoencephalography/electroencephalography, functional magnetic resonance imaging) and models, e.g. using representational similarity analysis. We showcase two recent efforts that have been undertaken in this spirit and provide novel results about visual and auditory scene analysis. Finally, we discuss the limits of this perspective and sketch a concrete roadmap for future research.

This article is part of the themed issue ‘Auditory and visual scene analysis’.

## 1. Introduction

Imagine walking through the marketplace in an old town square. You traverse the large open space, filled with stalls, ringed by distant buildings and dominated by a cathedral at one end. Passing groups of chatting shoppers, shouting vendors and singing birds, you enter the cathedral and find yourself in the dark entryway. Close grey walls surround you, and outside sounds are damped so that you only hear your own footsteps in the space. Your percepts change dramatically a third time as you proceed to the light-filled nave, your footsteps now reverberating from the high ceilings and walls. The instantaneous and effortless parsing of every scene in this everyday sequence belies the enormous computational complexity of this task. Ambiguous, noisy input—both visual and auditory—is rapidly converted into a percept of the spatial layout that can guide your navigation and distinguish meaningful objects [1–3]. Reflecting this complexity, the first few hundred milliseconds of processing a stimulus feature a large cascade of rapidly activated brain regions, transforming sensory representations at each step.

Understanding these spatio-temporal neural dynamics poses major methodological and conceptual challenges for contemporary cognitive neuroscience. We identify three major methodological stumbling blocks: the noisiness of current brain imaging data; the inherently sparse and nonlinear relationship between stimuli and neural response patterns; and the lack of non-invasive brain measurement techniques highly resolved in both time and space.

In this opinion piece, we suggest a tripartite approach as a remedy, addressing each stumbling block, respectively: multivariate pattern classification techniques, complex computational modelling inspired by computer science, and a common quantitative framework for combining different neuroimaging and modelling results. To elucidate the proposed approach, we summarize two recent research efforts investigating visual and auditory scene perception. Finally, we discuss limits of the proposed approach and suggest concrete examples for further research in visual and auditory scene perception along the presented methodological lines.

## 2. Three current methodological challenges in unravelling human visual and auditory scene perception

### (a) Brain signals measured non-invasively in humans are inherently noisy

The first challenge exemplifies the difficulty of *in vivo* physiological measurements. The neuroimaging methods in standard use throughout cognitive neuroscience are non-invasive and thus inherently noisier than direct neuronal recordings. The most common techniques are functional magnetic resonance imaging (fMRI) and electro- and magnetoencephalography (M/EEG). fMRI is sensitive to blood oxygenation levels [4], which correlate with local neuronal activity. Owing to thermal and physiological noise, signal changes from neuronal activity–related blood oxygenation typically amount to only a few per cent of the measured signal. While M/EEG measures neuronal activity more directly compared with fMRI, it is equally affected by instrumental, physiological (e.g. muscle artefacts from breathing and heartbeats) and environmental (e.g. all other electrical equipment in the vicinity) noise [5].

Future improvements in recording techniques, such as ultrahigh-field fMRI [6,7] and new types of MEG sensors [8], will probably continue to improve signal-to-noise (SNR) ratios. However, immediate benefits are available from current analysis techniques that make the best use of weak information in noisy brain data.

### (b) The sparseness and nonlinearity of neuronal responses obscures the computations underlying scene perception

The second challenge is a consequence of incomplete mechanistic information. Fully understanding a complex system—such as the neural systems underlying visual and auditory scene perception—requires a quantitative model of the neural computations involved. A common and principled approach is decomposition of the system into parts, and the stepwise sequential modelling of the computations in each part from bottom to top, e.g. from sensory input to the high-level representation of the scene [9].

However, for this approach to work, what is to be modelled must be known in quantitative and detailed terms: the brain regions involved in the computation and the neuronal response properties in those regions. These preconditions pose a problem. The regions involved in visual and auditory scene processing can be inferred from sources such as neuropsychology, anatomy and brain imaging, but this knowledge is incomplete [10]. Further, along the neural processing cascade, neurons respond increasingly sparsely and nonlinearly to sensory stimulation, making systematic investigation of detailed response properties difficult. In effect, for both visual and auditory processing, the bottom-up approach has been most successful in modelling early processing stages, and less so for higher processing stages in mid- and high-level cortical areas [9,11,12].

Further progress necessitates an alternative modelling approach to visual and auditory scene perception that does not depend on *a priori* detailed knowledge of neuronal response properties and step-by-step sequential discovery of processing stages from bottom to top.

### (c) No single non-invasive brain measurement technique provides a spatio-temporally integrated and algorithmically informed view of scene perception

The third challenge stems from the limitations of imaging modalities. Existing non-invasive brain measurement techniques commonly used in humans provide either high spatial or temporal resolution, but not both simultaneously. fMRI provides relatively high spatial resolution, typically on the order of a few millimetres or even below, but suffers from limited temporal resolution due to the sluggishness of the BOLD response [4]. M/EEG, by contrast, provides high temporal resolution at the level of single milliseconds, but suffers from comparably low spatial resolution [5,13]. Thus, for a spatio-temporally resolved view of brain function, information from several brain imaging techniques must be integrated [14–16] and related with modelling results, as described above for algorithmic information. However, there is no direct and easy mapping between sensor spaces in fMRI, MEG and computer models: it is difficult to determine how activity in any particular voxel would relate to activity in any particular MEG sensor or any particular model part. Thus, a principled alternative indirect approach is needed to quantitatively relate different brain measurements and models to each other.

## 3. A tripartite approach to tackle current methodological challenges

Here, we argue that progress can be made by a concerted effort based on three pillars of recent methodological development: (i) multivariate analysis techniques such as decoding and cross-decoding to increase sensitivity for low-SNR neuroimaging data; (ii) top-down modelling of brain activity by complex models—in particular, deep neural networks (DNNs)—to understand neuronal representations and algorithms; and (iii) the integration of imaging methods and models in a common quantitative framework using representational similarity analysis (RSA) [17]. Together these pillars support a common quantitative framework for a spatio-temporally resolved and algorithmically informed account visual and auditory scene perception. We describe the rationale of each methodological pillar below.

### (a) Multivariate pattern classification for noisy brain data

The overall goal of neuroimaging is to establish statistical dependencies between measurements of brain activity and experimental conditions. The received analysis approach towards this goal for M/EEG and fMRI is a mass *univariate* approach [18]: brain activity is measured with a large number of sensors, and then signal in each sensor is analysed *separately*. Activation differences between nearby sensors are assumed to stem from noise, motivating signal averaging across sensors to increase the SNR ratio. Thus, any signal in activation differences between sensors is lost, and only signal in mean activity is considered. Contrary to this expectation, it has emerged over the last decade that fine-grained differences between nearby sensors do contain valuable information, for both M/EEG and fMRI research on both visual and auditory perception [19–22]. Thus, instead of averaging across sensors, a *multivariate* approach that captures dependencies between activity in multiple sensors *in combination* and experimental conditions is called for.

A growing number of researchers in auditory and visual scene perception are making use of multivariate analysis methods (e.g. a small sample due to space constraints: [23–27]). We believe that further popularization of the multivariate analysis approach—for which a multitude of software toolboxes are readily available, e.g. [28,29]—will benefit future investigations of visual and auditory scene perception.

**Figure 1.**
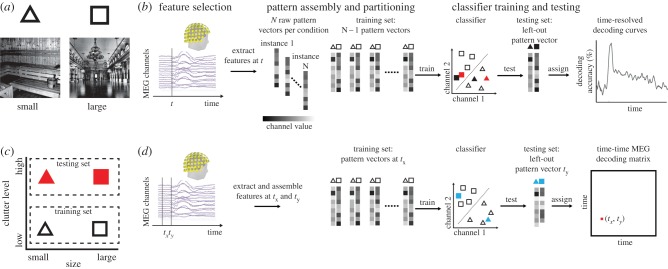
Illustration of the classification and cross-classification approach. (*a*) Participants are presented with two different experimental conditions, e.g. visual scenes differing in the real-world size shown (symbolized by triangle and square), while brain data (MEG) are recorded. (*b*) The time course is considered with respect to image onset. A four-step multivariate pattern-classification scheme analysis is used (following [19]). (i) *Feature selection*: Relevant sensors are selected as features. Here we consider all *M* sensors [30]. (ii) *Pattern assembly and partitioning*: Sensor patterns across the selected features are extracted from the measurements, assembled into pattern vectors of size 1 × *M* (where *M* = number of selected features), and labelled by the corresponding experimental condition. To avoid circularity [31] of subsequent analysis, patterns are partitioned into two independent sets: a *training data set* used to train a classifier and a *testing data set* to test the prediction of the trained classifier. (iii) *Classifier training*: A machine learning classifier is given the training set of pattern vectors (symbolized by outlined black triangles and squares) and the respective labels to learn a mapping from *M*-dimensional (shown for two dimensions for visualization) sensor activity to experimental conditions. That is, the classifier learns a decision boundary between classes (dotted line). (iv) *Classifier testing*: The trained classifier is fed the testing set (symbolized by filled black triangle and square) and is used to predict the labels (here: correctly). Correct performance of the classifier indicates that pattern vectors contain information about experimental conditions. Repeated for all time points, this four-step procedure results in a time course indicating the timing of dependencies between brain data and experimental conditions. (*c*) Cross-classification across conditions is a direct extension of the classification approach. Different conditions are assigned to the training and the testing set. For example, a classifier is trained on patterns for small versus large uncluttered scenes (black symbols) and is tested on patterns on cluttered scenes (red symbols, also in *b*). Correct classification indicates similarity of patterns and thus brain activity across the differences between the conditions in the training and testing set (scene size across clutter level). (*d*) Cross-classification across time. Here, brain data from different time points (e.g. *t_x_* and *t_y_*) is assigned to the training and the testing set. If repeated for all time-point combinations, this results in a time–time matrix, indicating similarities between patterns evoked by experimental conditions over time, and thus temporal stability of underlying neural representations. Figure adapted from [32].

Here we illustrate the general approach for the analysis of M/EEG data (for recent in-depth reviews focusing on fMRI, see [19–22]). The basic idea for establishing a statistical relationship between activity in multiple sensors and experimental conditions is to conceptualize activity in multiple sensors as patterns and to treat the analysis as a pattern classification problem. This turns the task into a straightforward application of pattern classification techniques readily available from machine learning (for detailed explanation, figure 1*a*). In short, an algorithm called a machine learning classifier learns a mapping between activation patterns and experimental conditions. Then, the classifier is tested on independent data. Successful prediction of experimental conditions by the classifier indicates the presence of information about experimental conditions in the activation patterns. This establishes a dependency between activation patterns and experimental conditions, which can subsequently be tested for statistical significance.

Classification reveals statistical dependencies between experimental conditions and brain activity, but does not characterize the neural representation further. In an extension of the classification approach known as cross-classification [33], different conditions are assigned to the training and testing sets (figure 1*c*). Correct cross-classification indicates similarity between the sensor patterns underlying the different conditions of the training and the testing set. This in turn is interpreted as tolerance of neural representations to whatever factors distinguish the conditions in the training and testing sets. For example, a classifier may be trained to distinguish between brain responses to animate and inanimate objects in a training set of images. High classification accuracy on brain responses to a second, independent (testing) set of images would be interpreted as sensitivity to the general property of animacy, rather than specific differences in the training set.

M/EEG, due to its high temporal resolution, offers the possibility of cross-classification across time [34] (figure 1*d*). For this, the classifier is trained on pattern vectors from one time point and tested on pattern vectors from other time points. Continued successful classifier performance is interpreted as evidence for temporal stability of the underlying neural representations, while transient neural representations would manifest in successful cross-classification only near the trained time point.

In sum, multivariate pattern analysis promises better detection power for weak signals in noisy brain data, allowing the investigation of questions in visual and auditory scene perception with stronger evidence basis, and helping to characterize the underlying neural computations both in the nature of the representations involved and their temporal dynamics.

### (b) Addressing challenge 2: top-down modelling to discover sensory representations

**Figure 2.**
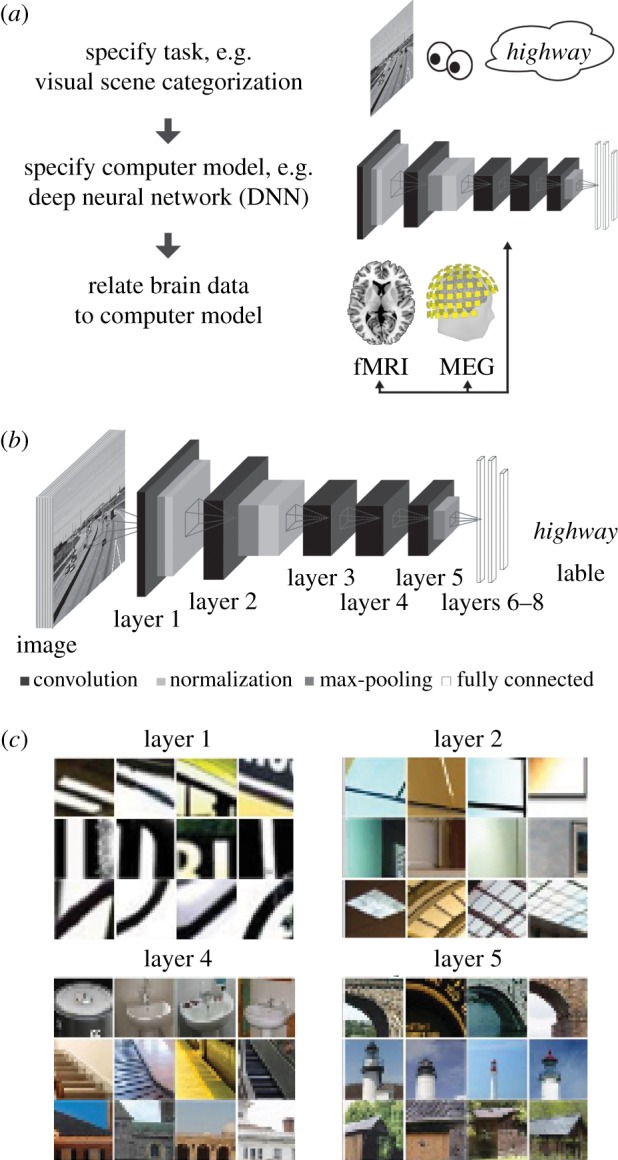
Illustration of the goal-driven complex modelling approach. (*a*) General approach. First, we specify a cognitive faculty to be explained, i.e. here the ability to classify scenes. Second, we define and train a computer model on the cognitive function of interest. Third, we record brain responses to stimuli with which both the model and the brain are probed. Finally, we compare the similarity of brain and the model responses to the stimuli. (*b*) DNNs as models of sensory processing. DNNs are a stack of layers (8) where on each layer a combination of convolution, normalization and max pooling is performed. The inputs are sensory data from the periphery (for vision: pixel values), and the output is defined by the task the DNN is trained on (labels for categorization). The network is initialized with random weights and learns features by supervised learning and gradient descent. (*c*) Visualization of neuron receptive fields by image fragments to which particular neurons are sensitive. The DNN learns useful features to carry out the task through training. Notably, the complexity of the features increases with layers, from simple Gabor-like selectivity to object parts and full objects. Figure adapted from [32,35].

An alternative to the bottom-up modelling approach for visual and auditory scene perception in cortex is to revert the direction of modelling to top-down (figure 2*a*) [36,37]. Thus, a specific objective of the brain becomes the starting point for the model: a cognitive task is defined, e.g. scene classification. Next, a computer model is trained to perform the specified objective. Finally, the model representations are compared with measured neural representations.

As we do not know *a priori* with which representations to model visual and auditory scene processing, models that learn the necessary representations themselves are of particular interest. A promising class of models are deep convolutional neural networks (DNNs) [24,25]. These multi-layered neural networks perform linear (convolution, i.e. point-wise multiplication with a filter) and nonlinear operations (pooling of responses across neurons, and thresholding) at each layer (figure 2*b*). When trained on tasks such as scene or object classification, these models learn the representations necessary to fulfil the task (figure 2*c*). DNNs perform better than any other known model class and sometimes even rival human performance [38].

Several studies using the top-down modelling approach have shown that DNNs employ similar visual representations as the brain. DNNs trained on visual object categorization explain more variance in high-level ventral visual cortex in monkey [39] and human [40] than any other model. Further, the relation between DNNs and the brain is hierarchical: representations in low-level visual areas were similar to lower layers of the network, and representations in high-level visual areas were similar to higher layers of the network [36,40,41]. A similar correspondence was found in processing time: higher layers of the DNN were similar to MEG data later in time with respect to image onset [36]. While the top-down modelling approach using DNNs was pioneered in vision, it is not limited to vision. DNNs perform well on auditory tasks, such as automated speech recognition [42,43]. Very recent research points towards a hierarchical relationship between processing stages of DNNs trained on auditory tasks and regions of the human auditory system [44,45]. This promises new insight on the much-debated delineation of functional sub-regions of auditory cortex and adds to the demonstrated utility of neural networks in parcellating not only speech but also the gist of auditory scenes (e.g. [46]). Fortunately, excellent software toolboxes to aid in DNN training are readily available [47,48].

Together, preliminary results suggest that top-down modelling of visual and auditory scene perception can provide valuable insight into the underlying neural architecture, algorithms and representations.

### (c) Addressing challenge 3: integration of imaging methods and models for a spatio-temporally resolved and algorithmically informed view

For a spatio-temporally integrated and algorithmically informed view on scene perception, brain measurements with techniques with high spatial resolution (such as fMRI), high temporal resolution (such as M/EEG) and models providing algorithmic explicitness must be combined in a common framework. A large body of research has established relations between all possible pairs of the triplet of M/EEG, fMRI and computational models: M/EEG and fMRI [15,49,50], fMRI and computational models [51–53] and M/EEG with computational models [32,54,55]. However, establishing a relation between any two members of a triplet is not informative about the relation to the third. Integrating all three members of the triplet in a common framework offers more comprehensive insight into the spatio-temporal dynamics of visual and auditory scene perception.

**Figure 3.**
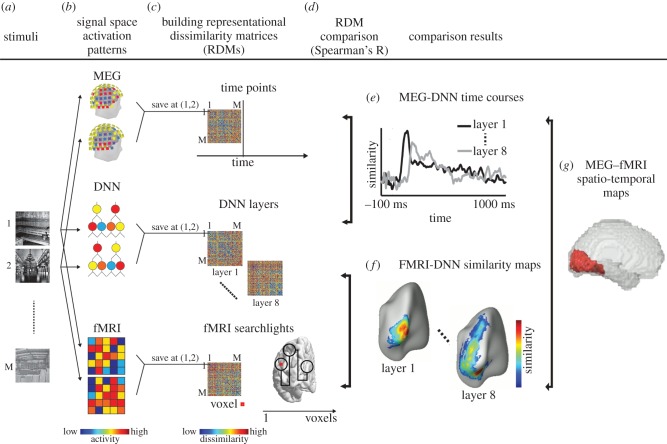
RSA as a quantitative framework for combining models and data from different neuroimaging techniques. (*a*) A large conditions set (scene images) is used to probe brain activation in different neuroimaging techniques and also computational models (DNNs). (*b*) Condition-specific patterns are recorded for each signal space. (*c*) To make data directly comparable, signal-space patterns are transformed into dissimilarity space. For this, all pairwise combinations of conditions are compared with each other by dissimilarity (e.g. by calculating 1 minus Spearman's correlation, or decoding accuracy of a classifier), forming so-called representational dissimilarity matrices (RDMs), indexed in rows and columns by the conditions compared. RDMs are a summary of the representational geometry of the signal space, indicating which conditions evoke similar or different patterns. Such RDMs can be formed for each time point with technique with high temporal resolution such as M/EEG, for each location in cortical space with techniques that have high spatial resolution, such as fMRI, and for parts of computational models, such as layers of the DNNs. (*d*) In the similarity space of RDMs, fMRI, M/EEG and models can be linked to each other, combining their respective advantages, by calculating the similarity between RDMs (e.g. by calculating simple Spearman's correlation between RDMs). This yields (*e*) MEG–DNN time courses, (*f*) MEG–fMRI spatio-temporal maps and (*g*) fMRI–DNN similarity maps. Figure adapted from [35].

A recent approach with this goal is representational similarity analysis (RSA) (for in-depth introduction and review, see [17,56]). The basic idea is to abstract away from the particular source spaces in which data is accrued, e.g. voxels, M/EEG sensors or model parts, into a common similarity space (figure 3*a*–*c*). The similarity space is defined by the similarity of patterns related to experimental conditions in the respective source space (voxels, sensors, etc.). Representational similarities are in the same space and can thus easily be linked directly to each other through second-order similarity (i.e. similarity of similarities, figure 3*d*). Thus, this approach has the potential to create links in a common, quantitative framework between any comparisons across disparate source spaces, e.g. M/EEG and fMRI to computational models (fig. 3*e*,*f*) [36,40] or to each other (figure 3*g*) [30,58]. Importantly, excellent toolboxes that ease the application of RSA in different programming environments are readily available [59,60].

Thus, the application of RSA to visual and auditory scene perception has the potential to bring together insights from different and usually disparate sources with synergistic gain. Note that RSA is open in principle to integration of any kind of additional information, such as data from different species [57] and behaviour [61]. In particular, it is well suited for the investigation of subject-specific idiosyncrasies in brain function beyond the group average [62,63]. By allowing the combination of behavioural measures with measures of neural data in a common framework, it assesses individual differences in a way unlikely to be affected by subject-specific differences unrelated to activity of the nervous system relevant for behaviour. In this way, subject-specific representations could augment analyses of individual differences by merging functional data with other neurophysiological [64] or behavioural [65–67] measures.

## 4. Highlighting two research efforts conducted in this spirit

In this section, we will highlight two recent research efforts in visual [32] and auditory [68] scene perception that make use of major parts of the tripartite, concerted method strategy, i.e. multivariate pattern classification, DNN modelling and integration of results in a quantitative framework.

Both studies investigate the question of spatial layout perception. Perceiving the layout of the visual scene is a crucial ability for all animals engaged in locomotion and navigation. Operationalizing spatial layout as the size of the space a scene subtends in the real world [27,69], the two studies investigated the temporal dynamics of visual and auditory scene perception, respectively.

### (a) The representation of the size of a visual scene in the human brain and deep neural networks

#### (i) Multivariate pattern classification reveals the time course of single-scene and scene size processing

**Figure 4.**
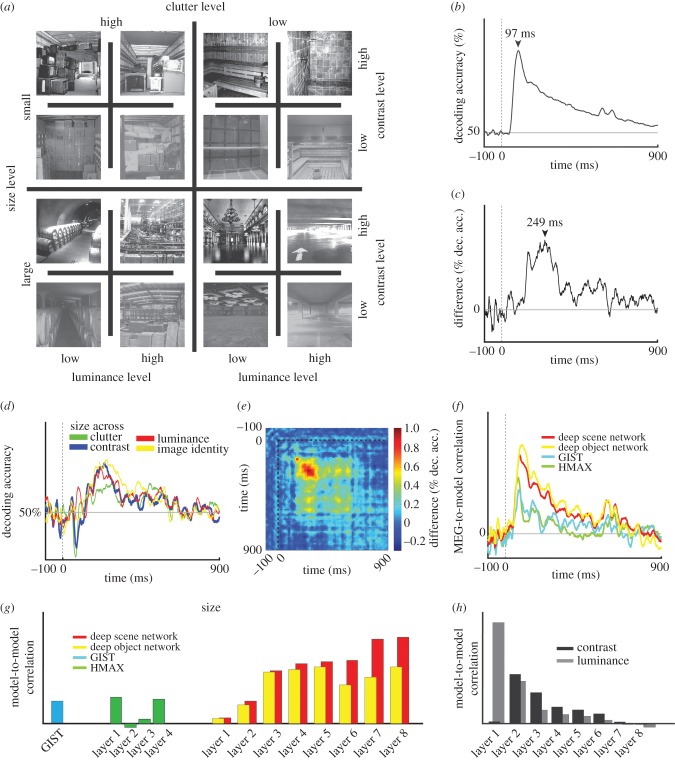
Dynamics in the visual representation of scene size. (*a*) The stimulus set consisted of 48 images, differing in four experimental factors (scene size, clutter level, contrast and luminance). (*b*) Results of single scenes classification from MEG data (50% chance level). Horizontal grey line indicates chance-level decoding. Vertical dotted line indicates stimulus onset. (*c*) Results of scene size classification (0% chance level). (*d*) Results of cross-classification of scene size across other experimental factors (50% chance level). (*e*) Results of cross-classification of scene size across time. (*f*) Comparison of representations in computational models to MEG data using RSA. (*g*) Scene size representations in computational models. (*h*) Representations of luminance and contrast in the DNNs. Stars in (*g*,*h*) indicate statistical significance at *p* < 0.05; FDR corrected for multiple comparisons. Figure adapted from [32].

To probe the representations of scenes in brains and computer models, Cichy *et al*. [32] used a stimulus set of 48 indoor images, differing along 4 orthogonal dimensions: scene size (large versus small), clutter level (high versus low clutter), luminance and contrast (figure 4*a*). In a first step, they used time-resolved multivariate pattern classification on MEG data (epoched from −100 to +900 ms w.r.t. stimulus onset) to identify the time course with which single images of scenes were discriminated by visual representations. This analysis revealed a robust signal with a peak at 97 ms (figure 4*b*). In a second step, they identified the part of the observed signal directly related to the representation of scene size. For this, they compared the results of the classification analysis for images of same and different scene size, subtracting the average of the former from the latter. They found that scene size was represented in the brain with a distinctive time course, peaking at approximately 249 ms (figure 4*c*).

#### (ii) Cross-classification reveals tolerance of visual scene size representations to other scene properties

To be ecologically useful, representations of scene size must be tolerant to variation in other properties of the scene, such as low-level image features arising from particular viewing conditions (e.g. luminance and contrast), and to other properties of scene such as the amount of clutter or category. To investigate the tolerance of scene representations, the authors used cross-classification across orthogonal experimental dimensions. For example, to investigate the tolerance of scene size representations to clutter level, they trained a classifier on MEG sensor patterns related to low-clutter scenes and tested the classifier on MEG sensor patterns related to high-clutter scenes. They found that the scene size signal was tolerant to changes in luminance, contrast, clutter and scene identity (figure 4*d*). Finally, to establish the temporal stability of representations underlying the representation of scene size, Cichy *et al*. used cross-classification across time. They found that scene size representations were stable over time from approximately 200 to 600 ms (figure 4*e*).

Together, these results showed the time course with which scene representations emerge in the human brain, exemplifying the potential of multivariate pattern analysis for understanding the neural mechanisms of visual scene processing.

#### (iii) Comparison to computational models suggests common mechanisms for the emergence of scene properties in brains and artificial networks

To investigate the nature of visual scene representations, the authors [32] compared scene representations in human brains with computational models. The set of computational models consisted of two standard models of scene and object perception (HMAX and GIST) [9,70] and two DNN models trained on object and scene categorization.

First, using RSA, the authors investigated how well each computational model accounted for emerging visual scene representations. They found that while all models had similar representations of scenes as the human brain, representations of the DNN models were most similar (figure 4*f*).

In a second step, the authors further investigated the emergence of scene size further in DNNs. The findings were threefold. First, both a DNN trained on object and on scene categorization predicted the scene size of an image (figure 4*g*). This indicates that DNNs capture abstract scene properties in their representations even when not being trained to do so, suggesting by analogy how scene size representations may emerge in neural circuits. Second, the representation of scene size increased with increasing layer number of the network, indicating the gradual build-up of representations that index scene size (figure 4*g*). Interestingly, the opposite was observed for low-level image properties, luminance and contrast (figure 4*h*). This suggests that DNNs have a brain-like hierarchical processing structure in which representations of relevant visual properties gradually emerge and representations of irrelevant visual properties of visual scenes are lost. Third, the DNN trained on scene categorization had stronger representations of scene size than the DNN trained on object categorization (figure 2*g*). This shows that the visual task on which DNNs are trained changes representational structure, concurrent with the presence of partly overlapping processing streams in the human brain for different visual contents such as objects and scenes [71,72].

Together, these results show how the nature of scene processing can be elucidated from a computational perspective, using complex computational models such as DNNs and comparison with brain data using RSA.

### (b) The representation of the size of an auditory scene

#### (i) Multivariate pattern classification reveals the time course of single-scene identity and scene size processing

**Figure 5.**
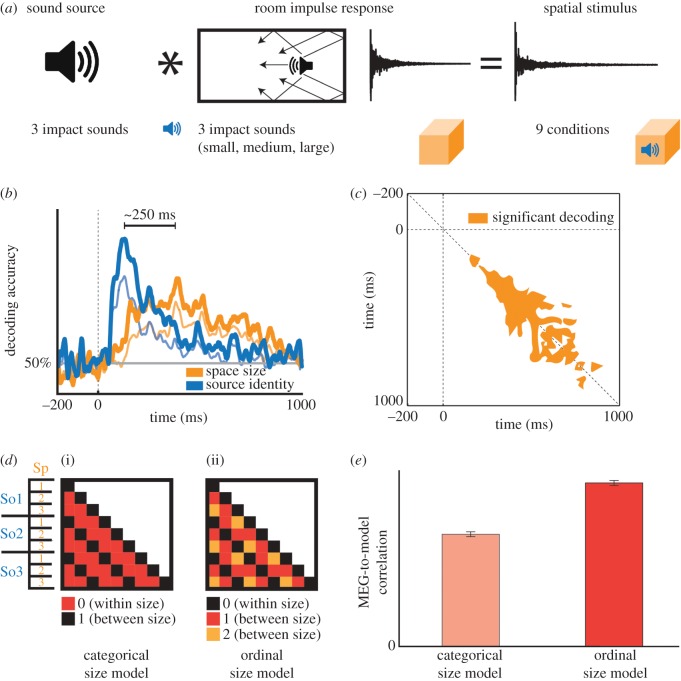
Dynamics in the auditory representation of scene size. (*a*) Stimulus set comprised nine auditory scenes, generated by convolving each of three brief impact sounds with each of three room impulse responses. (*b*) MEG classification analysis revealed dissociable trajectories of source identity (blue) and space size (orange) decoding, peaking approximately 250 ms apart. Cross-classification analysis revealed that source identity and space size decoding were robust across different spaces and sources, respectively. Chance level of classification was 50%. Bold traces: main classification decoding curves. Thin traces: cross-classification decoding curves. Horizontal grey line indicates chance-level decoding. Vertical dotted line indicates stimulus onset. (*c*) Temporal generalization analysis of space size decoding reveals dynamics of evolving representations, tending towards greater stability (width relative to diagonal) with increasing time. (*d*) Model RDMs hypothesizing a categorical (i) versus ordinally progressive (ii) representation of space size. (*e*) Correlation with MEG data reveals stronger relationship with the progressive model. Figure adapted from [68].

In a similar design applied to the auditory domain, Teng *et al*. [68] used a set of nine stimuli, varying across orthogonal dimensions of space size and sound source (figure 5*a*). Participants listened passively to the stimuli, responding with a button press to occasional deviant vigilance stimuli. The MEG data were epoched from −200 to 1000 ms relative to stimulus onset and were analysed to determine the time course of single-condition classification. The classification peak of this time course occurred at 156 ms post-stimulus onset.

The classification analysis was repeated with stimulus conditions pooled by space size or sound source identity, yielding two distinct time courses of sound source and space size discrimination, peaking approximately 250 ms apart (figure 5*b*). This suggests that the representations encoding spatial extent are separable in time, potentially drawing on different temporal features of the stimuli. In a control experiment, the authors report that decoding peaks occurred in a similar time window even when stimuli were equated at 2 s duration, implicating a consistent neural time course irrespective of stimulus duration. A follow-up experiment (not shown) confirmed that behavioural reaction times and accuracies of pairwise same-different judgments correlated significantly with MEG classification peak latencies and decoding accuracies, suggesting that the neurodynamic signal reflects perceptual processing.

#### (ii) Cross-classification across scene properties and time reveals stability of auditory representations

To test the stability of these representations, the authors cross-classified space size across sound sources (training space size on two sound sources, then testing on the third), and vice versa. The resulting time courses peaked at nearly identical time points as the pooled classification analysis, indicating that auditory scene attribute representations are tolerant to variations such as spectral and level differences (figure 5*b*). A temporal generalization analysis classifying across time points (figure 5*c*) revealed relatively rapid initial dynamics, indicated by classifier performance close to the diagonal, and increasing stability of the neural representation thereafter, indicated by a ‘spread’ of classifier performance to time points distant from the diagonal.

#### (iii) Model fits suggest progressive space size representation

To evaluate the nature of the auditory representations, the authors compared the MEG signal to simple models hypothesizing pure categorical or ordinal space size coding (figure 5*d*). These took the form of representational dissimilarity matrices (RDMs) in which each cell denoted a categorical difference between space sizes (representational distance of 0 versus 1) or a stepwise progression (0, 1 or 2). The ordinal model was found to correlate more strongly with the MEG data than the categorical model (figure 5*e*), suggesting that the neural representations are more consistent with a sequential progression of space size coding than with a generic categorical distinction; i.e. that space is represented neurally along a size dimension [27,69].

## 5. Limits of the proposed methodological approach

The goal of this opinion piece is to encourage use of advanced analysis methods in the study of visual and auditory scene perception. The immediate limits of this perspective are thus the respective limits of the proposed methods.

For classification and cross-classification, the major limitation is that these methods do not explicitly model how the brain implements, i.e. realizes the probed representation. Instead, the nature of the specific neural realization has to be inferred. To add this missing link, pattern classification approaches can be complemented with encoding models [73].

Relatedly, the major drawback of RSA is the direct flipside of its major advantage: the relation of representations to specific realizations is not specified, i.e. many different realizations can have the same similarity structure. On the one hand, this enables comparison across disparate spaces; on the other, it leaves an unspecific link between representation and realization. To rephrase this in the framework of David Marr's levels of analysis of a cognitive system [74], the levels of analysis show some independence, evident in the fact that one algorithm or representation can be implemented (i.e. realized [75]) in many different ways. However, not all realizations may be on par once processing speed and efficiency are assessed, or the performing system's error profile is evaluated [74]. Thus, further investigation is needed to fully reveal the neuronal realization of representations in the human brain.

The major drawback of top-down models is that they are not explicit about the same aspects as bottom-up models. In particular, bottom-up models are explicit about the nature of the representations in the model, whereas top-down models are not. Thus, additional techniques must be applied to those models to visualize and understand features (e.g. [76]). For DNNs as the most widely used models for top-down modelling today, in particular, two limits are that massive supervised learning is not a realistic learning scheme for the brain and that the brain's perceptual functions include much more than categorical identification of stimuli. Approaches using unsupervised and one-shot learning might be able to narrow this gap [77].

## 6. Concrete roadmap for further research

We believe that the presented framework may enrich any type of research in visual and cognitive scene processing. Here we exemplify this potential in a concrete roadmap for further research to three open questions in scene processing.

First, what are the spatio-temporal dynamics of visual and auditory scene processing, respectively? Previous research has focused on revealing either the temporal or the spatial dimension. Integrated methods such as fMRI–M/EEG fusion [30,58] might yield insight into how the information flows in the network of regions related to scene processing in the visual [2] and auditory [1] cortical networks and their interaction [78]. Revealing the order of, and thus input–output relationships between, the nodes of the scene-processing cortical network will aid understanding of scene processing at the representational and algorithmic levels [74].

Second, what are the spatio-temporal dynamics with which information from the visual and auditory modalities are integrated to yield a unified percept of the scene [79]? The research efforts sketched above revealed the time course with which scene size representations emerge in vision and audition separately, but did not relate those to each other. It is an exciting venue for further research using RSA and cross-classification methods on both fMRI and MEG data to reveal where and when in the brain modality independent scene properties emerge in the brain.

Third, what is the detailed nature of visual and auditory scene representations along the processing hierarchy in each modality? DNN modelling and top-down comparison with neural data are well suited to investigate this question. For example, it has recently been shown that task-orthogonal object properties—such as object size, position and pose—emerge along the hierarchy of a DNN trained on visual object classification, rather than being abstracted away [37]. Strikingly, this was found to mirror the coding of the ventral visual stream in humans, and thus suggested re-thinking our understanding about where different types of visual object information is represented in the human brain. This directly motivates further research into the nature of representation of scene properties such as spatial layout, the contained objects, functional role and affordances in both the visual and the auditory domain. Further, while the current fit between human brains and DNNs is stunning, it might be further improved, e.g. by increasing architectural similarity [80]. In particular, one possibility is to build a fovea–periphery organization as present in human retina into the DNN and to evaluate its consequences [81]. A specific consequence of this might be the emergence of a correspondence in topography between DNNs and brains. In the human brain, it has been observed that face-selective regions have a foveal bias and scene-selective regions have a peripheral bias [82,83]. Introducing a fovea–periphery organization into the DNN might lead to the emergence of a similar topography in the DNN.

Finally, in this article, our discussion and detailed examples have predominantly featured examples of sensory processing of scenes and their features. However, at a given moment, only fragments of scenes are accessible to consciousness, guided and filtered by attention [46], salience [84] and task demands. The strength and flexibility of our approach—accessing and relating fine-grained representations in a variety of data modalities—can also be applied here, offering an additional tool to pursue elusive signals such as the auditory neural correlate of consciousness (NCC_A_, [85]).

## 7. Conclusion

In sum, we have argued for a tripartite effort to further understanding of the neural mechanisms underlying visual and auditory scene perception: multivariate analysis methods, an integrated quantitative framework and top-down computational modelling. Acknowledging that theory and elegant experiments cannot be supplanted by analysis methods, we are convinced that the latter opens new horizons and opportunities not to be missed by the contemporary investigator.
